# Are Systemic Manifestations Ascribable to COPD in Smokers? A Structural Equation Modeling Approach

**DOI:** 10.1038/s41598-018-26766-x

**Published:** 2018-06-05

**Authors:** Laurent Boyer, Sylvie Bastuji-Garin, Christos Chouaid, Bruno Housset, Philippe Le Corvoisier, Geneviève Derumeaux, Jorge Boczkowski, Bernard Maitre, Serge Adnot, Etienne Audureau

**Affiliations:** 1 APHP, Hôpital Henri Mondor, Département de Physiologie-Explorations Fonctionnelles, DHU A-TVB, F-94010 Créteil, France; 2 INSERM U955 and Université Paris Est (UPEC), UMR U955, Faculté de médecine, Créteil, F-94010 France; 3 APHP, Hôpital Henri Mondor, Département de Santé Publique, F-94010 Créteil, France; 4 Université Paris Est (UPEC), Faculté de médecine, CEpiA, EA7376, Créteil, F-94010 France; 5 Centre Hospitalier Intercommunal, Département de Pneumologie et Pathologie Professionnelle, Créteil, F-94000 France; 6 Inserm, Centre d’Investigation Clinique 1430, and APHP, Hôpital Henri Mondor, F-94010 Créteil, France

## Abstract

Whether the systemic manifestations observed in Chronic Obstructive Pulmonary Disease (COPD) are ascribable to lung dysfunction or direct effects of smoking is in debate. Structural Equations Modeling (SEM), a causal-oriented statistical approach, could help unraveling the pathways involved, by enabling estimation of direct and indirect associations between variables. The objectives of the study was to investigate the relative impact of smoking and COPD on systemic manifestations, inflammation and telomere length. In 292 individuals (103 women; 97 smokers with COPD, 96 smokers without COPD, 99 non-smokers), we used SEM to explore the pathways between smoking (pack-years), lung disease (FEV_1_, K_CO_), and the following parameters: arterial stiffness (aortic pulse wave velocity, PWV), bone mineral density (BMD), appendicular skeletal muscle mass (ASMM), grip strength, insulin resistance (HOMA-IR), creatinine clearance (eGFR), blood leukocyte telomere length and inflammatory markers (Luminex assay). All models were adjusted on age and gender. Latent variables were created for systemic inflammation (inflammatory markers) and musculoskeletal parameters (ASMM, grip strength, BMD). SEM showed that most effects of smoking were indirectly mediated by lung dysfunction: e.g. via FEV_1_ on musculoskeletal factor, eGFR, HOMA-IR, PWV, telomere length, CRP, white blood cells count (WBC) and inflammation factor, and via K_CO_ on musculoskeletal factor, eGFR and PWV. Direct effects of smoking were limited to CRP and WBC. Models had excellent fit. In conclusion, SEM highlighted the major role of COPD in the occurrence of systemic manifestations while smoking effects were mostly mediated by lung function.

## Introduction

Chronic obstructive pulmonary disease (COPD) develops in 30% of smokers and is characterized by airflow limitation and alveolar destruction or emphysema. This disease is a major health concern, causing considerable morbidity and mortality^1^. Although initially described as a lung disease, extra pulmonary chronic conditions, or comorbidities, play a major role in disease course and contribute mainly to the severity of the disease and impact its prognosis and mortality^2–4^. The history of these systemic manifestations remains unclear in the development of the disease.

COPD and not directly to smoking, based on observational studies showing an increased prevalence of comorbidities in COPD patients compared to smokers without COPD and non-smokers^5–9^. Van Remoortel *et al*., has challenged this concept by comparing newly diagnosed COPD patients to smokers without COPD and non-smokers^10^. They have shown that smoking history and reduced daily physical activity may be the main risk factor for development of comorbid conditions, reactivating the debate of the respective role of lung alteration and smoking in systemic manifestations. This raises the question whether these systemic alterations and COPD are related to a common pathogenic mechanism initiated by smoking or whether systemic manifestations are directly driven and/or worsened by lung alterations. Excess of inflammation or a process of premature aging were proposed as mechanism linking all these phenomenons^11,12^.

While strict causal inference remains out of reach of observational studies, advanced approaches may help better differentiating direct and indirect determinants – then called mediators – of systemic manifestations in smokers. For that matter, Structural Equation Modeling (SEM) is a suitable method that proved useful to unravel multidirectional associations and potential causal pathways in complex diseases or pathologic syndromes^13,14^. Yet, no study has addressed the issue of the development of systemic manifestations in smokers and COPD patients using such an approach. Finally, because smoking and lung alterations may gradually exert their potential systemic effects within a continuum, relevant associations may be overlooked when only focusing on clinically recognized diseases. There is a need to investigate these complex associations using continuous biological and functional parameters operating also at earlier stages of disease development.

The objective of this study was to propose a comprehensive model based on SEM that integrates the interrelationships between cigarette smoke exposure, lung alterations associated with COPD and systemic manifestations of the disease. Our hypothesis was that aging-related parameters and systemic manifestations are both affected directly by smoking and indirectly (mediated) via the alteration of the respiratory parameters associated with COPD. To do so, we used objectively measured parameters such as arterial stiffness, bone mineral density, muscle mass, insulin resistance and kidney function to investigate the mediating pathways from cigarette smoke exposure to systemic manifestations.

## Methods

### Study population and data collection

We studied 292 participants including 97 smokers with COPD, 96 smokers without COPD, and 99 non-smokers recruited at the Henri-Mondor Teaching Hospital between January 2009 and September 2012. The characteristics of the three groups were describe previously^5^. The study was approved by the institutional review board of the Henri-Mondor Teaching Hospital (CPP, #09–027). All participants provided written informed consent before inclusion. All methods were performed in accordance with the relevant guidelines and regulations.

Patients with clinically stable COPD were recruited prospectively at the pulmonology outpatient clinic and potential smokers without COPD at the smoking-cessation clinic and clinical investigations center. Non-smokers were healthy volunteers recruited from the general population by the clinical investigation center of the Henri-Mondor Teaching Hospital. A patient was considered as smoker when his history of tobacco smoking was higher than 10–pack-year. They were evaluated clinically before study inclusion. Subjects could have moderate to severe comorbidities, to the exception of a chronic heart failure (LEVF <45%), active malignancy, or inflammatory systemic diseases (i.e. rheumatoid arthritis, lupus, spondyloarthritis).

Arterial stiffness (aortic pulse wave velocity, PWV), bone mineral density (BMD), appendicular skeletal muscle mass (ASMM), grip strength, insulin resistance (HOMA-IR), creatinine clearance (eGFR), blood leukocyte telomere length and cytokines (Luminex assay) were measured in each subjects (see supplementary material for detailed methodology).

### Conceptual framework

As recommended when applying SEM methodology, we defined a priori a conceptual framework of the relationships between variables^15^. We hypothesized that systemic manifestations are both affected directly by smoking and indirectly (mediated) via the alteration of the respiratory function parameters associated with COPD. Specifically, we used Forced expiratory volume in 1 second (FEV_1_) and diffusing capacity for carbon monoxide corrected for alveolar volume (K_CO_) to assess lung function and modeled systemic manifestations as continuous parameters based on previously reported evidence regarding their association with COPD, including bone mineral density, appendicular muscle mass index and pinch/grip tests to assess musculoskeletal disorders^8,16^, pulse-wave velocity to assess arterial stiffness^17^, glomerular filtration rate to assess kidney dysfunction^18^, telomere length to assess premature ageing^19^, CRP, white blood cells count (WBC) and cytokines (MCP-1, TNFα, IL-6 and IL-8) as measurements of systemic inflammation^20,21^.

### Statistical analysis

We used structural equation modeling to formally evaluate interdependent relationships among the three sets of variables, i.e. smoking pack years, lung function parameters, and systemic manifestations. In a nutshell, SEM is a statistical method based on factor analysis and linear regression modeling used to test how well a prespecified model actually fit observed data. SEM enables to examine complex relationships between variables, including the estimation of direct and indirect associations through mediating pathways. Modeling can use observed variables (indicators) and unobserved latent variables (factors) estimated from several indicators. The hypothesized model is represented in path diagrams where circles represent factors and rectangles represent indicators. Alleged causal effects are plotted as single-headed arrows, while bidirectional correlations are represented with double-headed curved arrows. Results are reported as standardized coefficients to facilitate interpretation of the estimates. Standardized coefficients range from -1 (indicating a completely negative relationship) to 1 (indicating a completely positive relationship) and allow the comparison of the relative strength of their values across the model. On the basis of the pre-specified conceptual framework, the following groups of observed variables were combined into latent variables (factors): a ‘musculoskeletal factor’ (ASMMI, hip and lumbar BMD, pinch and grip tests) and a ‘cytokines factor’ (IL-6, IL-8, TNFα, MCP-1).

The main analysis was led on the whole sample (Model 1). Two alternative models were estimated to assess the robustness of the modeling, considering only smokers and COPD patients (Model 2) to assess the impact of null smoking pack years values in non-smokers, and after creating a latent variable based on FEV_1_ and K_CO_ to model lung function parameters as a global pulmonary factor instead of two separate features (Model 3). All analyses were systematically adjusted on age and gender. Paths not statistically significant at the p < 0.05 level were removed from the model and path diagram.

The following standard SEM adequacy fit indices were used to assess model goodness of fit: the comparative fit index (CFI; >0.90 considered as adequate), the Tucker-Lewis index (TLI; >0.90 considered as adequate), the root mean square error of approximation (RMSEA; ≤0.05 good, 0.05–0.08 adequate, >0.08 poor) along with its 90% confidence interval (CI) as is conventional in SEM analyses (lower CI limit close to 0 and upper limit <0.08 considered adequate)^22,23^. Modification indices were considered for applying minor changes to the three pre-specified models to improve goodness of fit (e.g. by adding or removing relationships).

Descriptive results are given as percentages for categorical data, and means (±standard deviation [SD]) or medians (interquartile range [IQR]) for continuous variables. Variables were analyzed using either raw values (age, gender, smoking pack years, FEV1 and KCO) or log-transformed values (all other parameters), depending on the normality of their distribution as assessed graphically and by the means of the Shapiro-Wilk test.Pearson’s correlation coefficients (r) were computed to assess the bivariate associations between candidate factors. For illustrative purpose, a correlation network plot was built from those results and a principal component analysis (PCA) was performed to construct a Gabriel’s biplot projecting the subjects along the principal components axes, based on their own individual characteristics^24^.

A p-value < 0.05 was considered significant. Estimations were based on the maximum likelihood with missing values (MLMV) method using STATA v14.2 (StataCorp, College Station, TX, USA) and R statistical software (3.3.0) was used for correlation analyses and visualizations (qgraph, pca3d and ggplot2 packages).

### Ethics approval

The study was approved by the institutional review board of the Henri-Mondor Teaching Hospital (CPP, # 09–027). All participants provided written informed consent before inclusion. All methods were performed in accordance with the relevant guidelines and regulations.

## Results

### General subjects characteristics

The study population consisted of 292 subjects recruited between 2009 and 2012. Clinical and biological characteristics of the subjects are detailed in Table 1. Mean age was 59.4 years (±7.3 years), and 35% of the subjects were females. Among them, 33% (97 subjects) were smokers with COPD, 33% (96 subjects) smokers without COPD and 34% (99 subjects) non-smokers.

**Table 1:** Main characteristics of the study population, N = 292.

	N completed	
Age, years	292	59.4 (±7.3)
Gender, women (%)	292	103 (35.3%)
Smokers	292	193 (66.1%)
Pack-years in smokers	186	42.6 (±24.8)
COPD, n (%)	292	97 (33.2%)
BMI, Kg/m²	292	25.7 (±4.1)
Obesity (BMI ≥ 30 Kg/m²), n (%)	292	38 (13.0%)
**Pulmonary function parameters**		
FEV1, % predicted	291	87.2 (±29.3)
FEV1/FVC	289	70.7 (±16.2)
KCO, % predicted	242	83.0 (±20.9)
**Systemic manifestations**		
BMD total lumbar, g/cm²	288	1.10 (0.98;1.20)
BMD hip (lowest), g/cm²	289	0.95 (0.85;1.06)
Osteoporosis, n (%)	267	38 (14.2%)
Pinch test, Kg	242	6 (5;8)
Grip test, Kg	242	37 (26;45)
ASMMI, Kg/m²	284	7.4 (6.3;8.3)
Sarcopenia, n (%)	283	30 (10.6%)
Glomerular flow rate, mL/min	267	88.4 (72.6;101.1)
HOMA-IR	278	1.93 (1.17;2.81)
Diabetes, n (%)	285	14 (4.9%)
Obliterans arteritis, n (%)	285	9 (3.2%)
Myocardial infarction, n (%)	285	12 (4.2%)
**Biological parameters**		
Telomere length (T/S) ratio	265	0.41 (0.35;0.48)
WBC count, Giga/l	270	6.30 (5.20;7.70)
CRP, mg/l	269	1.20 (0.40;5.00)
IL-6, pg/ml	264	15.6 (13.2;18.1)
IL-8, pg/ml	264	46.4 (40.3;51.7)
MCP-1, pg/ml	264	36.3 (27.5;47.8)
TNF-alpha, pg/ml	264	68.1 (56.6;82.1)

Results are given as means (±standard deviation) or medians (interquartile range), unless otherwise stated.

*Definition of abbreviations:* % predicted, percentage of the predicted value; BMI, body mass index; KCO, transfer factor coefficient of the lung for carbon monoxide; BMD, bone mineral density; ASMMI, appendicular skeletal muscle mass index; HOMA-IR, homeostatic model assessment of insulin resistance; T/S, ratio of telomere-repeat copy number over single-gene copy number, WBC, white blood cells; Glomerular flow rate was estimated using the Cockcroft-Gault formula.

### Structural equation modeling

**Figure 1:**
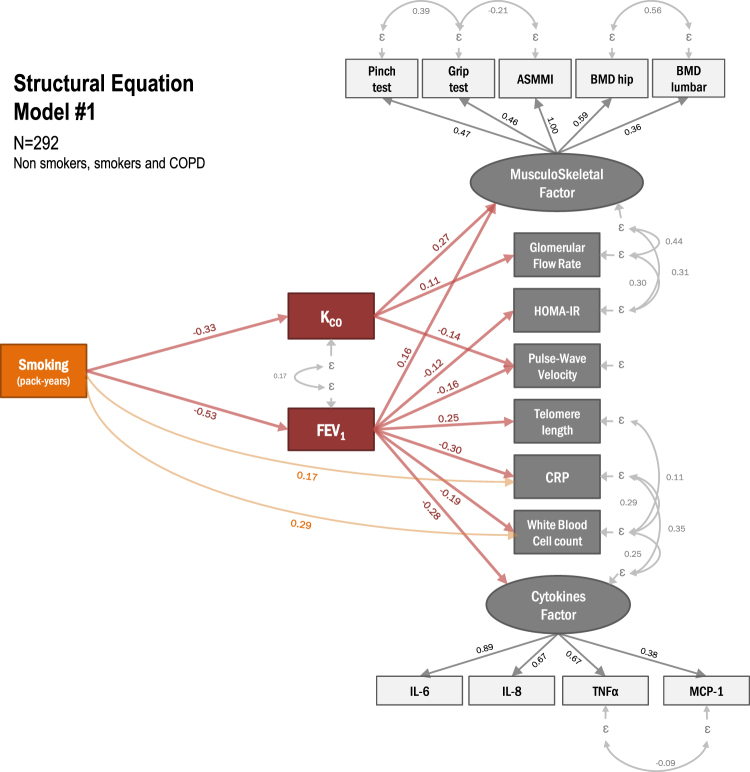
Structural equation model with pathways from cigarette smoke exposure to systemic manifestations (*Model 1;* N = 292 non-smokers, smokers and COPD patients). Variables in circles are unobserved (latent) factors explaining observed (manifest) variables in rectangles. Arrows indicate the hypothesized pathways with numbers as the standardized regression coefficients of direct effects after adjusting on age and gender. All shown effects are statistically significant at the p < 0.05 level. FEV1: forced expiratory volume in 1 s; KCO, transfer factor coefficient of the lung for carbon monoxide; BMD, bone mineral density; ASMMI, appendicular skeletal muscle mass index; HOMA-IR, homeostatic model assessment of insulin resistance.

We used SEM to investigate whether aging-related parameters were affected *directly* by smoking and/or *indirectly* (mediated) via the alteration of the respiratory function parameters associated with COPD.,

Results from the first model conducted on the whole sample (N = 292) is shown in Fig. 1, with standardized coefficients for the pathways involving FEV_1_, K_CO_ and smoking pack years detailed in Table 1. Significant paths were identified from smoking pack years to FEV_1_ and K_CO_. SEM revealed in addition that the effects of cigarette smoke on systemic manifestations and telomere length were mainly mediated by lung alteration, as indicated by the very limited number of direct links towards systemic manifestations, i.e. only found for CRP and WBC. In contrast, statistically significant pathways were found via FEV_1_ towards musculoskeletal parameters, HOMA-IR, arterial stiffness as assessed by PWV, telomere length, CRP, WBC and inflammation, and via KCO towards musculoskeletal parameters, creatinine clearance and arterial stiffness. Model 1 demonstrated very good fit based on the RMSEA = 0.050, CFI = 0.942 and TLI = 0.922 (Table 2).

**Table 2:** Main results from structural equations modeling: standardized coefficients of smoking and pulmonary parameters on ageing parameters.

	Model 1*			Model 2			Model 3	
	Smoking (PY)	FEV1, %	KCO, %	Smoking (PY)	FEV1, %	KCO, %	Smoking (PY)	Pulmonary factor
	Std. coeff.	Std. coeff.	Std. coeff.	Std. coeff.	Std. coeff.	Std. coeff.	Std. coeff.	Std. coeff.
Muskuloskeletal factor	NS	**0**.**162**	**0**.**270**	NS	**0**.**209**	**0**.**256**	NS	**−0**.**263**
Glomerular flow rate, mL/min	**0**.**15**	NS	**0**.**113**	NS	NS	NS	NS	**0**.**165**
HOMA-IR	NS	**−0**.**122**	NS	NS	NS	NS	NS	**0**.**187**
Pulse-wave velocity, m/s	NS	**−0**.**157**	**−0**.**144**	NS	**−0**.**244**	NS	NS	**0**.**285**
Telomere length (T/S) ratio	NS	**0**.**254**	NS	NS	**0**.**273**	NS	**−0**.**189**	NS
WBC count, Giga/l	**0**.**288**	**−0**.**199**	NS	**0**.**183**	**−0**.**144**	NS	NS	**0**.**529**
CRP, mg/l	**0**.**173**	**−0**.**295**	NS	**0**.**141**	**−0**.**315**	NS	NS	**0**.**528**
Cytokines factor	NS	**−0**.**278**	NS	NS	**−0**.**275**	NS	NS	**0**.**449**
**Goodness of fit indices**								
RMSEA (90% CI)	0.050 (0.039; 0.061)			0.043 (0.027; 0.058)			0.057 (0.047; 0.067)	
CFI	0.942			0.950			0.922	
TLI	0.922			0.936			0.900	

*Models 1, 2 and 3 are illustrated in Fig. 1, Supplemental Figs 1 and 2, respectively.

All models adjusted on age and gender.

Definition of abbreviations: NS, statistically not significant at the p < 0.05 level; Std. Coeff, Standardized regression coefficient; CFI, comparative fit index; TLI, Tucker-Lewis index; RMSEA, root mean square error of approximation; CI: confidence interval; HOMA-IR, homeostatic model assessment of insulin resistance; T/S, ratio of telomere-repeat copy number over single-gene copy number, WBC, white blood cells.

To test the stability of our results, we also conducted two additional analyses of sensibility under alternative study population or modeling approach. First, we built a SEM using a sample population restricted to smokers and COPD patients, thus excluding subjects with null pack years values (Model 2). Doing so yielded results very similar to those obtained on the whole sample (Figure E1), retrieving most previously identified pathways involving pulmonary parameters and pack years and equally satisfying goodness of fit indices (Table 2). Three pathways were not statistically significant in this modified analysis (i.e. from K_CO_ to PWV, from K_CO_ to eGFR and from FEV_1_ to HOMA-IR), an observation likely to reflect the lowered statistical power due to restricted sample size. Second, we performed a SEM on the whole sample but combining FEV_1_ and K_CO_ into a common pulmonary factor (Model 3). All pathways originating from this were found statistically significant, to the exception of the relation telomere length only directly affected by smoking (Figure E2). Goodness of fit indices were slightly inferior using this approach (Table 2).

### Correlation analyses

**Figure 2:**
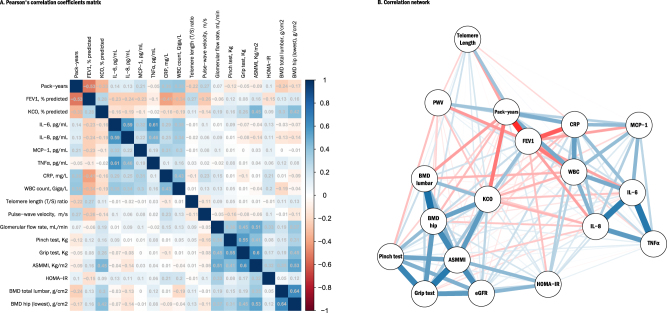
Correlation between aging-related parameters. (**A**) Pearson’s correlation coefficients matrix and (**B**) Correlation network. (**A**) The matrix contains the Pearson’s correlation coefficients between smoking pack-years, pulmonary function parameters and the 15 aging-related parameters of interest. Colors indicate the direction and the strength of the correlation, with positive correlations being displayed as blue tones and negative ones as red tones. (**B**) The correlation network is constructed from all pairwise correlations between items in (**A**). Items are represented by nodes and are connected by edges. Red and blue lines represent negative and positive correlations, respectively. Line width color saturation is proportional to the strength of the correlation.

Correlations between parameters are shown in Fig. 2 panel A (correlation matrix) and panel B (correlation network), demonstrating the strong correlations between cigarette smoke exposure and lung function parameters, between musculoskeletal parameters and between markers of systemic inflammation, respectively. Biplot visualizations based on principal component analysis are shown in Figure E3 (2-D plot**)** and Video E4 (3-D plot), illustrating the differentiated phenotypes across the three groups, with non-smokers generally projecting on the left part of the plot, smokers and more markedly patients with COPD gradually projecting on the right, indicating decreasing (worsening) pulmonary function and musculoskeletal parameters and increasing inflammation markers. Additional details are given in supplemental material.

## Discussion

The present study sought to determine direct and indirect effects of cigarette smoke on systemic manifestations of COPD disease using for the first time a SEM approach. Our analysis has demonstrated the non-straightforward relationships between cigarette smoke, lung determinants and arterial stiffness, bone mineral density, muscle mass, insulin-resistance, or renal function. The model showed good fit with the data and highlight several features: i) smoking effects were mostly mediated by lung function, underlining the major role of COPD and lung alteration in the occurrence of systemic manifestations ii) significant direct effects of smoking were strictly limited to inflammatory markers (CRP, White Blood Cells count) iii) the model did not show any direct or indirect effects of inflammation or telomere length on systemic manifestations.

No direct effect of cigarette smoke exposure on systemic manifestations was observed in our study; lung function alterations were the most important mediators of cigarette smoke effects on systemic manifestations. COPD is nowadays considered as a complex and multicomponent disease affecting not only the lung, but also associated with systemic manifestations leading to comorbidities, such as cardiovascular disease, osteoporosis, diabetes. These systemic manifestations play a pivotal role in the course of the disease and have a key impact on health care use, hospitalization and mortality in this population^3,4,9^. However whether these comorbidities are ascribable to cigarette smoke directly or COPD remains to be defined. Results of studies differ depending on their design^6,10,25^. Addressing the question of direct and indirect effects of smoking remains of crucial importance for the clinical care of COPD patients: demonstrating the specific role of COPD in the development of comorbidities among smokers would lead to more active screening strategies and treatment of comorbid conditions in COPD patients.

One strength of the present study lies in the original methodology to determine the complex origins of systemic manifestations in smokers with or without COPD. The relationship between cigarette smoke and several comorbidities such as cardiovascular diseases, osteoporosis, sarcopenia or kidney diseases is well documented and several studies are in favor of a pivotal role of COPD in this process^6,8,26–30^. However, the issue of the respective role of COPD and smoking has mainly been addressed so far by drawing comparisons between COPD patients, smokers without COPD and non-smokers, using unadjusted or standard multivariate analysis to quantify average relationships between factors. Advanced approaches such as clusters analysis or comorbidities network^25,31,32^ have provided useful descriptions on how lung alteration and comorbidities may combine in COPD populations, but do not inform on the causal interrelations at play. Alternatively, causality-oriented approaches such as SEM allow to test more complex models involving direct and indirect pathways, enabling the quantification and comparison of the relative strength of the associations. SEM does not unequivocally demonstrate causal relationships but still proves very useful to verify whether a conceptual model is actually supported by the data.

Although most of the effects of smoking are mediated by lung alteration in this population, our results also emphasis the heterogeneous ways linking cigarette smoke exposure, lung alterations and systemic manifestations. Indeed, smoking effects were different depending of the mediation through KCO or FEV1. Cigarette smoke in COPD patients causes a series of pulmonary manifestations, with variable combinations of alveolar damage such as emphysema leading to alveolar diffusion alterations, and bronchial remodeling leading to increased resistance of the respiratory tract. Lower bone density, muscle mass reduction and arterial stiffness have been frequently linked to the emphysema phenotype, independently of FEV_1_ level^8,28,33^. We also observed a direct link of K_CO_ to these systemic manifestations. However, except for kidney function, a direct link was also documented with FEV1 for bone density, muscle mass and function or arterial stiffness, suggesting, as other reports, that alteration of lung diffusion did not match systematically with emphysema in smokers and may correspond to a different pattern of physiologic abnormalities^34^. Similarly, the effect of smoking on inflammatory parameters and telomere length were mediated by FEV_1_, but not lung diffusion, confirming the multicomponent aspect of cigarette smoke induced lung alteration on extra-pulmonary manifestations.

Our results provide important insight on the development of COPD comorbidities: One of the main hypothesis to explain this association is the link between systemic inflammation and lung alteration of COPD^35^. Comorbidity of COPD and systemic inflammation may be the “overspill” result of inflammatory mediators released from the lung into the circulation^36^. This hypothesis is supported by previous studies showing that various inflammatory mediators are released from the aging lung or from the emphysematous lung as part of the “senescence associated secretory phenotype” of lung cells^37–41^. Indeed, in smokers, the lung is the first tissue impacted by cell senescence, a quiescent state of cell resulting in many changes including secretory^37^. In analogy, changes in the ability of adipose tissue to release cytokines and adipokines have profound pathophysiological impacts^42^, and some of these changes are now shown to be related to cell senescence during aging^43^. However, although we found a direct effect of lung alterations on the systemic inflammation in favor of the overspill hypothesis, we were not able to show a direct link between inflammation and the different parameters of systemic manifestations. Another hypothesis is that small inhaled particles that are part of the cigarette smoke may diffuse more easily from the airways to the circulation when the lung structure is altered. This phenomenon could explain the potentialization of systemic effect of cigarette smoke when the lung is altered.

In the present study, systemic manifestations were carefully and objectively quantified and their evaluation were not based on self-reported data^31^. The model used in this study showed significant relationship between each systemic manifestations, in particular between muscle mass and bone mineral density, insulin resistance or renal function. However, arterial stiffness as a strong marker of cardiovascular risk, was not associated to this manifestations. This is in contrast with some observations of vascular stiffness and osteoporosis in COPD patients^6^. However, this observation was not confirmed by studies describing COPD phenotype using clusters analysis^25,31^. Cardiovascular, cachectic or metabolic clusters appeared as distinct clusters confirming our hypothesis by a different approach. One of the underlying mechanisms of these systemic manifestations may be a process of accelerated aging affecting differently the organs and induced by the lung disease. Bone mineral density and muscle mass or function decline, decrease of glomerular filtration, increase of arterial stiffness and insulin resistance are usual aspects of the aging process. However, no direct link was observed between telomere length and age related systemic manifestations, suggesting that additional mechanisms are needed in association with telomere length reduction to drive the systemic manifestations. Altogether, these elements may suggest that mechanism linking the lung disease and systemic manifestations differed depending of the manifestation, whether it is low bone mineral density, sarcopenia or cardiovascular disease. Further longitudinal studies describing the course of the systemic manifestations are needed to confirm this hypothesis.

The main strength of our study is the development of a multidimensional model that integrates the contribution of multiple factors that influence systemic manifestations during COPD or cigarette smoke exposure. However some limitations of the study must be carefully considered. This study is limited by its cross-sectional rather than prospective design. Relatedly, while SEM is suitable to test the statistical plausibility of a pre-specified model given the observed data, it does not prove the existence of causal relationships. No causal inference can be formally proposed, and our model should be interpreted from an association point of view. Future longitudinal studies are needed to establish whether the model is longitudinally robust and whether lung alterations precede the onset of the comorbidities, fact that could confirm the major impact of lung alterations in the development of multi-organ disease that affects smokers.

## Conclusion

This study contributes to a better understanding of the relationship between cigarette smoke exposure, lung alteration of COPD and the development of aging related systemic manifestations. Although smoking cessation remains of prime importance to reduce lung and systemic manifestation or comorbidities, our study emphasis the strong link between lung alterations and comorbidities and the necessity of developing treatment that targets simultaneously COPD and its systemic manifestations. Determining whether treatment of COPD improves comorbidities and reciprocally whether treatments of comorbidities improves COPD remain crucial to improve the health status of COPD patients.

## Electronic supplementary material

Supplementary Information

Biplot of variables and observations from principle component analysis

## References

[1] Vogelmeier CF (2017). Global Strategy for the Diagnosis, Management, and Prevention of Chronic Obstructive Lung Disease 2017 Report. GOLD Executive Summary. American journal of respiratory and critical care medicine.

[2] Barnes PJ, Celli BR (2009). Systemic manifestations and comorbidities of COPD. Eur Respir J.

[3] Sin DD, Anthonisen NR, Soriano JB, Agusti AG (2006). Mortality in COPD: Role of comorbidities. Eur Respir J.

[4] Divo M (2012). Comorbidities and risk of mortality in patients with chronic obstructive pulmonary disease. American journal of respiratory and critical care medicine.

[5] Boyer L (2015). Aging-related systemic manifestations in COPD patients and cigarette smokers. PLoS One.

[6] Sabit R (2007). Arterial stiffness and osteoporosis in chronic obstructive pulmonary disease. American journal of respiratory and critical care medicine.

[7] Houben-Wilke S (2017). Peripheral Artery Disease and Its Clinical Relevance in Patients with Chronic Obstructive Pulmonary Disease in the COPD and Systemic Consequences-Comorbidities Network Study. American journal of respiratory and critical care medicine.

[8] Bon J (2011). Radiographic emphysema predicts low bone mineral density in a tobacco-exposed cohort. American journal of respiratory and critical care medicine.

[9] Mannino DM, Thorn D, Swensen A, Holguin F (2008). Prevalence and outcomes of diabetes, hypertension and cardiovascular disease in COPD. Eur Respir J.

[10] Van Remoortel H (2013). Risk factors and comorbidities in the preclinical stages of chronic obstructive pulmonary disease. American journal of respiratory and critical care medicine.

[11] Celli, B. R. *et al*. Inflammatory biomarkers improve clinical prediction of mortality in chronic obstructive pulmonary disease. *American journal of respiratory and critical care medicine* **185**, 1065–1072, 10.1164/rccm.201110-1792OC. 10.1164/rccm.201110-1792OC 22427534

[12] Agusti A (2012). Persistent systemic inflammation is associated with poor clinical outcomes in COPD: a novel phenotype. PLoS One.

[13] Spruyt K, Gozal D (2012). A mediation model linking body weight, cognition, and sleep-disordered breathing. American journal of respiratory and critical care medicine.

[14] Chih AH (2016). Mediating pathways from central obesity to childhood asthma: a population-based longitudinal study. Eur Respir J.

[15] Penke, L. & Deary, I. J. Some guidelines for structural equation modelling in cognitive neuroscience: the case of Charlton *et al.’s* study on white matter integrity and cognitive ageing. *Neurobiol Aging* **31**, 1656–1660; discussion 1561–1656, 10.1016/j.neurobiolaging.2009.10.019 (2010). 10.1016/j.neurobiolaging.2009.10.019 20079555

[16] Ogura-Tomomatsu H (2012). Predictors of osteoporosis and vertebral fractures in patients presenting with moderate-to-severe chronic obstructive lung disease. COPD.

[17] Wang LY (2017). Subclinical atherosclerosis risk markers in patients with chronic obstructive pulmonary disease: A systematic review and meta-analysis. Respir Med.

[18] Fedeli U (2017). Lung and kidney: a dangerous liaison? A population-based cohort study in COPD patients in Italy. Int J Chron Obstruct Pulmon Dis.

[19] Savale L (2009). Shortened telomeres in circulating leukocytes of patients with chronic obstructive pulmonary disease. American journal of respiratory and critical care medicine.

[20] Chaouat A (2009). Role for interleukin-6 in COPD-related pulmonary hypertension. Chest.

[21] Liu SF, Chin CH, Wang CC, Lin MC (2009). Correlation between serum biomarkers and BODE index in patients with stable COPD. Respirology.

[22] Weston R, Gore PA (2006). A brief guide to structural equation modeling. The counseling psychologist.

[23] Hooper D, Coughlan J, Mullen M (2008). Structural equation modelling: Guidelines for determining model fit. The Electronic Journal of Business Research Methods.

[24] KR G (1971). The biplot graphic display of matrices with application to principal component analysis. Biometrika.

[25] Burgel PR (2010). Clinical COPD phenotypes: a novel approach using principal component and cluster analyses. Eur Respir J.

[26] Degens H, Gayan-Ramirez G, van Hees HW (2015). Smoking-induced skeletal muscle dysfunction: from evidence to mechanisms. American journal of respiratory and critical care medicine.

[27] Swallow EB (2007). Quadriceps strength predicts mortality in patients with moderate to severe chronic obstructive pulmonary disease. Thorax.

[28] Chandra D (2012). The relationship between pulmonary emphysema and kidney function in smokers. Chest.

[29] Incalzi RA (2010). Chronic renal failure: a neglected comorbidity of COPD. Chest.

[30] Polverino F (2017). A Pilot Study Linking Endothelial Injury in Lungs and Kidneys in Chronic Obstructive Pulmonary Disease. American journal of respiratory and critical care medicine.

[31] Vanfleteren LE (2013). Clusters of comorbidities based on validated objective measurements and systemic inflammation in patients with chronic obstructive pulmonary disease. American journal of respiratory and critical care medicine.

[32] Divo MJ (2015). COPD comorbidities network. Eur Respir J.

[33] McAllister DA (2007). Arterial stiffness is independently associated with emphysema severity in patients with chronic obstructive pulmonary disease. American journal of respiratory and critical care medicine.

[34] Alcaide AB (2017). Clinical Features of Smokers With Radiological Emphysema But Without Airway Limitation. Chest.

[35] Sin DD, Man SF (2006). Skeletal muscle weakness, reduced exercise tolerance, and COPD: is systemic inflammation the missing link?. Thorax.

[36] Sinden NJ, Stockley RA (2010). Systemic inflammation and comorbidity in COPD: a result of ‘overspill’ of inflammatory mediators from the lungs? Review of the evidence. Thorax.

[37] Adnot S (2015). Telomere Dysfunction and Cell Senescence in Chronic Lung Diseases: Therapeutic Potential. Pharmacology & therapeutics.

[38] Dagouassat M (2013). The cyclooxygenase-2-prostaglandin E2 pathway maintains senescence of chronic obstructive pulmonary disease fibroblasts. American journal of respiratory and critical care medicine.

[39] Noureddine H (2011). Pulmonary artery smooth muscle cell senescence is a pathogenic mechanism for pulmonary hypertension in chronic lung disease. Circ Res.

[40] Hashimoto M (2016). Elimination ofp19(ARF)-expressing cells enhances pulmonary function in mice. JCI Insight.

[41] Houssaini, A. *et al*. mTOR pathway activation drives lung cell senescence and emphysema. *JCI Insight* **3**, 10.1172/jci.insight.93203 (2018). 10.1172/jci.insight.93203 PMC5821218 29415880

[42] Fasshauer M, Bluher M (2015). Adipokines in health and disease. Trends Pharmacol Sci.

[43] Schosserer M, Grillari J, Wolfrum C, Scheideler M (2018). Age-Induced Changes in White, Brite, and Brown Adipose Depots: A Mini-Review. Gerontology.

