# Supplementary material for: Are Systemic Manifestations Ascribable to COPD in Smokers? A Structural Equation Modeling Approach
Source: Sci Rep. 2018 Jun 5;8:8569. doi: 10.1038/s41598-018-26766-x (PMC5988713; doi:10.1038/s41598-018-26766-x)
Supplement: Supplementary file 1 — Supplementary Information [file 41598_2018_26766_MOESM1_ESM.docx]

**Are Systemic Manifestations Ascribable to COPD in Smokers? A Structural Equation Modeling Approach**

**Supplementary informations**

Laurent Boyer ^1, 2^, Sylvie Bastuji-Garin ^3,4^, Christos Chouaid ^5^, Bruno Housset ^5^, Philippe Le Corvoisier ^6^, Geneviève Derumeaux ^1,2^, Jorge Boczkowski ^2^, Bernard Maitre ^5^, Serge Adnot ^1,2^, Etienne Audureau ^3,4,*^

^1^APHP, Hôpital Henri Mondor, Département de Physiologie-Explorations Fonctionnelles, DHU A-TVB, F-94010, Créteil, France;

^2^INSERM U955 and Université Paris Est (UPEC), UMR U955, Faculté de médecine, Créteil, F-94010, France;

^3^APHP, Hôpital Henri Mondor, Département de Santé Publique, F-94010, Créteil, France;

^4^Université Paris Est (UPEC), Faculté de médecine, CEpiA, EA7376, Créteil, F-94010, France;

^5^Centre Hospitalier Intercommunal, Département de Pneumologie et Pathologie Professionnelle, Créteil, F-94000, France;

^6^ Inserm, Centre d’Investigation Clinique 1430, and APHP, Hôpital Henri Mondor, F-94010, Créteil, France France

^*^ etienne.audureau@aphp.fr

**Results**

**Figure 2: panel A** (correlation matrix) and **panel B** (correlation network)

Increasing cigarette smoke exposure was strongly correlated with decreasing respiratory function parameters (FEV_1_ and K_CO_), as indicated by the negative correlation coefficients between pack-years and FEV_1_ (r=-0.53) and K_CO_ (r=-0.33) and the close proximity of those variables in the corresponding correlation network. musculoskeletal parameters were found to be closely correlated, including lumbar and hip BMD, ASMMI, and results from pinch and grip test. Of note, eGFR was also correlated with these parameters as well as, to a lesser extent, K_CO_, whereas correlations with smoking pack years and FEV1 were found only moderate to poor. A third group of features demonstrated high correlations, involving biological measurements relating to inflammation, i.e. cytokines, CRP and white blood cells count. The two latter markers were particularly correlated with smoking pack years and FEV_1_, while associations with K_CO_ were less apparent. Telomere length and pulse-wave velocity were found somewhat isolated in the correlation network, detecting only slightly moderate correlations with smoking pack-years and FEV_1_.

**Online figure E3** and **online Video E4**

Based on these correlations, we then conducted a principle component analysis to construct a Biplot visualization that combines both observations (subjects) and variables in a common space, thus facilitating interpretation of the relationships identified (**online figure E3** and **online Video E4**). Dots corresponding to subjects were colored differently based on 3 pre-specified groups according to smoking profile, i.e. non-smokers (N=99), smokers without COPD (N=96) and patients with COPD (N=97). While non-smokers generally projected on the left part of the plot, smokers and more markedly patients with COPD gradually projected on the right, indicating decreasing (worsening) pulmonary function and musculoskeletal parameters and increasing inflammation markers.

**Methods**

**Measurements**

*Lung function*

Each participant underwent spirometry, plethysmography, and DL_CO_ measurement according to ATS/ERS consensus guidelines^1^. DL_CO_ and K_CO_ were corrected for hemoglobin.

*Assessment of systemic manifestations*

Arterial stiffness (aortic pulse-wave velocity, PWV) was measured as carotid-femoral pulse-wave velocity using Complior Analyse (Alam Medical, Vincennes, France). Bone mineral density (BMD) at the hip (femoral neck) and lumbar spine was determined using dual-energy X-ray absorptiometry (Lunar iDXA™, GE Healthcare, UK). Appendicular skeletal muscle mass index (ASMMI) was measured as fat-free soft tissue masses of legs and arms divided by height squared^2^. Pinch and grip strengths were measured using a standard handgrip dynamometer and pinch gauge (Baseline Evaluation Instruments, NY, USA), insulin resistance by calculating HOMA-IR (insulin·glucose)/22.5), and renal function by estimating the glomerular filtration rate (eGFR) using the Cockcroft-Gault formula.

*Blood tests*

Bead-based cytometric immunoassay (Bio-Rad Laboratories, Hercules, CA) was used to quantify blood circulating levels of IL-6, IL-8, MCP-1 and TNF-α.. Telomere length was assessed using real-time quantitative polymerase chain reaction (RT-qPCR) ^3^.

**References**

1 Miller, M. R. *et al.* General considerations for lung function testing. *Eur Respir J* **26**, 153-161, doi:10.1183/09031936.05.00034505 (2005).

2 Baumgartner, R. N. *et al.* Epidemiology of sarcopenia among the elderly in New Mexico. *American journal of epidemiology* **147**, 755-763 (1998).

3 Savale, L. *et al.* Shortened telomeres in circulating leukocytes of patients with chronic obstructive pulmonary disease. *American journal of respiratory and critical care medicine* **179**, 566-571, doi:200809-1398OC [pii]10.1164/rccm.200809-1398OC (2009).

**Figure Legends**

**Online Figure E1** Structural equation model with pathways from cigarette smoke exposure to systemic manifestations in smokers (Model 2; N=193).

Variables in circles are unobserved (latent) factors explaining observed (manifest) variables in rectangles. Arrows indicate the hypothesized pathways with numbers as the standardized regression coefficients of direct effects after adjusting on age and gender. All shown effects are statistically significant at the p<0.05 level.

FEV1: forced expiratory volume in 1 s; KCO, transfer factor coefficient of the lung for carbon monoxide; BMD, bone mineral density; ASMMI, appendicular skeletal muscle mass index; HOMA-IR, homeostatic model assessment of insulin resistance.

**Online Figure E2** Structural equation model with pathways from cigarette smoke exposure to systemic manifestations through a pulmonary factor (Model 3; N=292).

Variables in circles are unobserved (latent) factors explaining observed (manifest) variables in rectangles. Arrows indicate the hypothesized pathways with numbers as the standardized regression coefficients of direct effects after adjusting on age and gender. All shown effects are statistically significant at the p<0.05 level.

FEV1: forced expiratory volume in 1 s; KCO, transfer factor coefficient of the lung for carbon monoxide; BMD, bone mineral density; ASMMI, appendicular skeletal muscle mass index; HOMA-IR, homeostatic model assessment of insulin resistance.

**Online Figure E3**. Biplot of variables and observations from principle component analysis

The Biplot representation visualizes the relationships between variables (arrows), while simultaneously displaying the patients (dots) based on their own individual characteristics. Results are projected onto the two first dimensions yielded by Principle Component Analysis (PCA). Colors for observations correspond to 3 pre-specified groups according to past smoking and COPD diagnosis, i.e. non-smokers (N=99), smokers (N=96) and patients with COPD (N=97), and demonstrate an apparent partitioning between subgroups.

**Online video E4**. Biplot of variables and observations from principle component analysis

The Biplot representation is here shown as a tridimensional and animated representation (see Figure 2 for a two-dimensional static representation), to better display the relationships between variables (arrows), while simultaneously showing the patients (dots) based on their own individual characteristics. Results are projected onto the three first dimensions yielded by Principle Component Analysis (PCA). Colors for observations correspond to 3 pre-specified groups according to past smoking and COPD diagnosis, i.e. non-smokers (N=99), smokers (N=96) and patients with COPD (N=97), and demonstrate an apparent partitioning between subgroups.

**Figure E1**


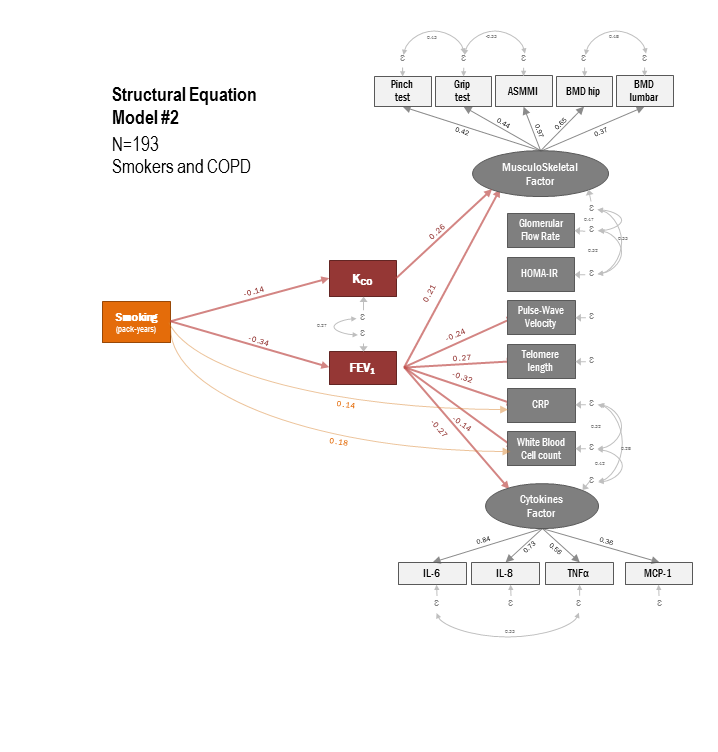


**Figure E2**

**
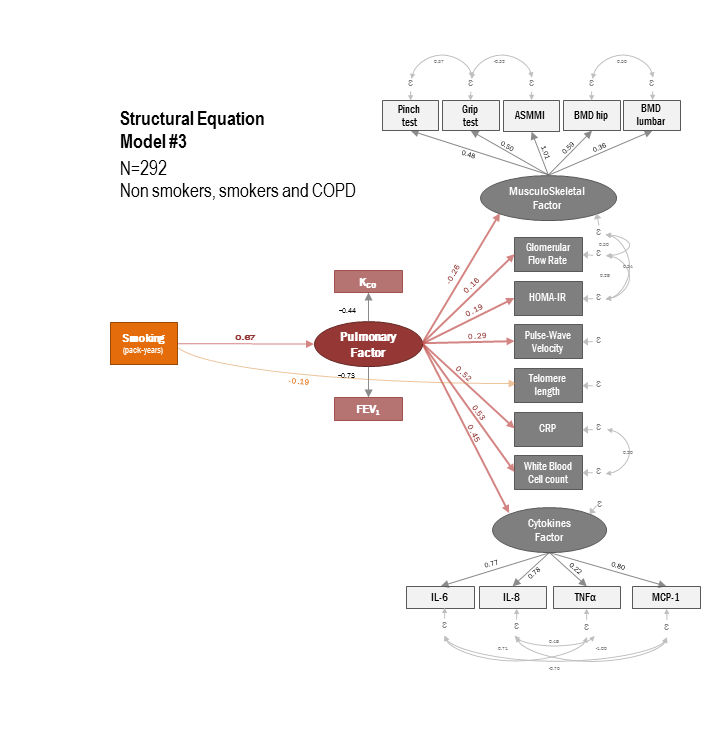
**

**Figure E3**

**
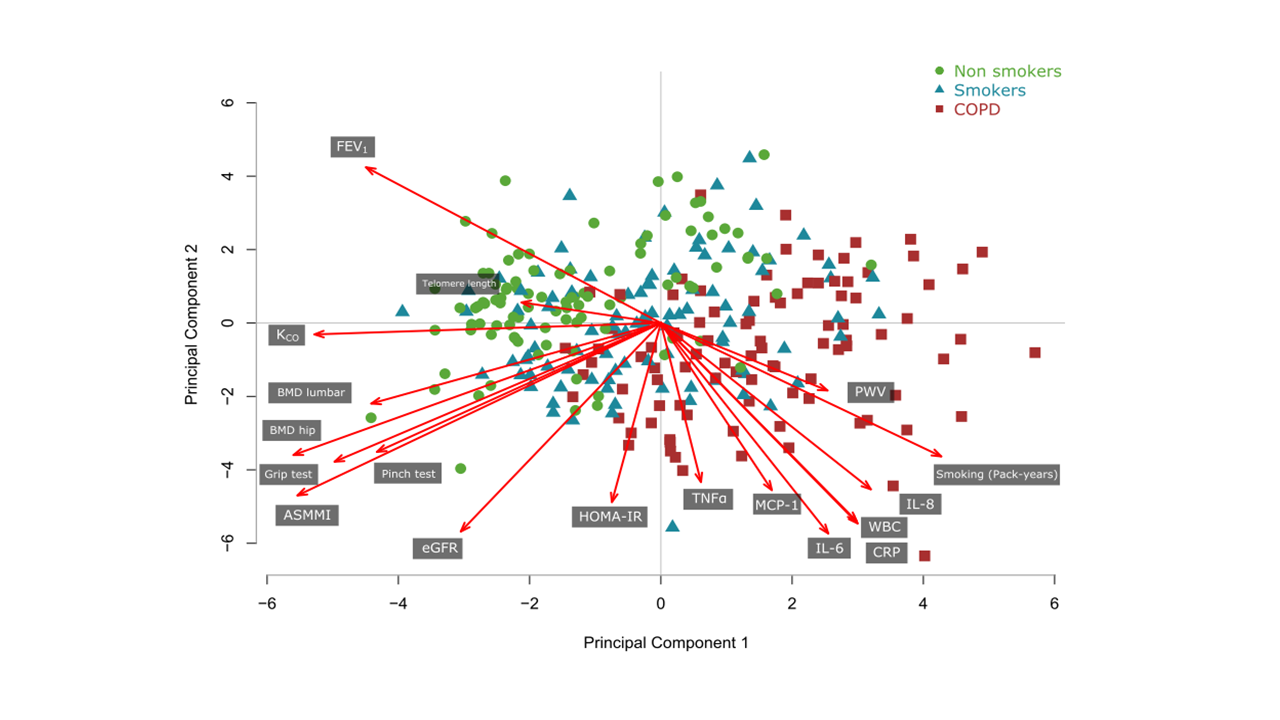
**
